# Evaluation of Acute Kidney Injury in Postcardiotomy Cardiogenic Shock Patients Supported by Extracorporeal Membrane Oxygenation

**DOI:** 10.31083/j.rcm2403091

**Published:** 2023-03-16

**Authors:** Jiachen Qi, Weidong Yan, Gang Liu, Yuan Teng, Sizhe Gao, Shujie Yan, Jian Wang, Boyi Zhou, Bingyang Ji

**Affiliations:** ^1^Department of Cardiopulmonary Bypass, Fuwai Hospital, National Center for Cardiovascular Disease, State Key Laboratory of Cardiovascular Medicine, Chinese Academy of Medical Science & Peking Union Medical College, 100037 Beijing, China; ^2^Department of Pain Medicine, Beijing Tsinghua Changgung Hospital, School of Clinical Medicine, Tsinghua University, 102218 Beijing, China

**Keywords:** extracorporeal membrane oxygenation, postcardiotomy cardiogenic shock, acute kidney injury, incidence, risk factor

## Abstract

**Background:**

This study sought to evaluate the incidence of acute kidney injury (AKI) defined by the Kidney Disease: Improving Global Outcomes (KDIGO) group in patients supported by veno-arterial extracorporeal membrane oxygenation (VA ECMO) after post-cardiotomy cardiogenic shock (PCS), and to identify the risk factors for AKI ≥3.

**Methods:**

Patients with and without AKI ≥3 were divided into two groups. Potential risk factors for developing AKI ≥3 were evaluated by univariate logistic regression analysis. Patient risk factors (*p *< 0.1) in the univariate analysis were entered into the multivariable logistic regression model. The tolerance and variance inflation factors (VIF) were calculated to evaluate the collinearity of the potential variables.

**Results:**

136 patients with a mean age of 53.6 ± 13.9 years (66.9% male) were enrolled in the study. 80 patients (58.8%) developed AKI ≥3. Patients with AKI ≥3 required significantly longer mechanical ventilation (200.9 [128.0, 534.8] hours *vs.* 78.9 [13.0, 233.0] hours, *p *< 0.001). The ICU stay and hospital stay of patients with AKI ≥3 were much longer than patients with AKI <3 (384 [182, 648] hours *vs.* 216 [48, 456] hours, *p* = 0.001; 25.0 [15.3, 46.6] days *vs.* 13.4 [7.4, 38.4] days, *p* = 0.022, respectively). There was no difference in preoperative risk factors between the two groups. Age, cross-clamp time, cardiopulmonary bypass (CPB) time, the timing of ECMO implantation, mean artery pressure (MAP), lactate concentration before ECMO, and preoperative ejection fraction (EF) were entered into the multivariable analysis. The timing of ECMO implantation was an independent risk factor for AKI ≥3 (*p* = 0.036). Intraoperatively implantation of ECMO may decrease the incidence of AKI ≥3 (odds ratio (OR) = 0.298, 95% confidence interval (CI) = 0.096–0.925). The tolerance and variance inflation factors showed that there was no collinearity among these variables.

**Conclusions:**

The incidence of AKI ≥3 in patients supported by VA ECMO after PCS was 58.8% in our center. Patients with AKI ≥3 required significantly longer mechanical ventilation and hospital stay. Intraoperative implantation VA ECMO was associated with a decreased incidence of AKI ≥3.

## 1. Introduction

Post-cardiotomy cardiogenic shock (PCS) in cardiac surgery is associated with a survival rate of only 25–44% [1]. Veno-arterial extracorporeal membrane oxygenation (VA ECMO) can be a life-saving procedure for temporary mechanical circulatory support, and its use in PCS patients has increased in recent years [2]. Although VA ECMO provides a period for cardiac recovery, complications and mortality remain significant. The survival rate has fluctuated between 20.8% and 65.4% amongst various centers with complications including strokes, acute kidney injury (AKI), bleeding, and thrombotic events [3, 4, 5]. AKI requiring continuous renal replacement therapy (CRRT) is particularly common in patients with PCS supported with VA ECMO, which negatively effects survival [3, 5, 6]. Identifying and managing risk factors for AKI will help decrease the incidence of AKI in this population.

It has been reported that earlier implantation of VA ECMO may help prevent the occurrence (65.7%) of stage 3 AKI defined by the Kidney Disease: Improving Global Outcomes (KDIGO) group in France [7]. The duration of low cardiac output associated with PCS may determine organ dysfunction. Because ethnic differences and the heterogeneity in ECMO management in different centers, we sought to evaluate the incidence and outcomes of AKI ≥3 in our center during ECMO following PCS. Therefore, the aim of this study was to report the incidence of AKI ≥3 in patients with PCS supported by VA ECMO, to investigate the prognostic impact of AKI ≥3, and to identify those risk factors for developing AKI ≥3.

## 2. Materials and Methods

### 2.1 Study Design

This observational, single-center respective study, was approved by the Fuwai Hospital Ethics Committee (2021-1496). Due to the retrospective design of this study, signed informed consent was waived. All patient data were anonymized.

### 2.2 Study Population

Patients 18 years and older undergoing a single VA ECMO run for PCS between January 2009 and December 2020 were included. The exclusion criteria were: (1) heart transplantation patients; (2) patients with preoperative ECMO or undergoing more than one ECMO run; and (3) patients with chronic hemodialysis.

### 2.3 ECMO Management

The decision for implantation or weaning of VA ECMO was decided by the ECMO management team, which consisted of a cardiac surgeon, intensive care specialist, and a perfusionist. Nearly 90% of peripheral cannulations were performed percutaneously (through the femoral vein and artery), which allows for chest closure and can reduce mediastinal bleeding. Central aortic cannulation was performed through the sternum with the cannulas already in place for cardiopulmonary bypass (CPB) for the patients who developed an aortic dissection or pulmonary dysfunction. The anticoagulation management of VA ECMO is summarized in the **Supplementary Files**. The goal after VA ECMO support was to reduce intravenous inotropes to allow optimal myocardial recovery. Cardiopulmonary recovery was assessed daily by clinical, echocardiographic, and hemodynamic measurements to define the optimal weaning time.

### 2.4 Groups and Endpoints

The primary endpoint was the incidence of AKI ≥3 in patients with PCS supported by VA ECMO and its impact on the postoperative outcomes. Patients with and without AKI ≥3 were divided into two groups. The outcomes evaluated included in-hospital death, septicemia, liver dysfunction, pneumonia, cerebrovascular accidents, atrial fibrillation, and malignant arrhythmias. In addition, we sought to identify the risk factors for developing AKI ≥3 using patient demographic data, the Charlson Comorbidity index, laboratory tests prior to ECMO, the type of surgery, intraoperative factors, and the time of ECMO implantation.

### 2.5 Definitions

AKI was defined according to the KDIGO guideline. The definition of AKI ≥3 was the stage 3 AKI evaluated by the KDIGO guideline within 7 days of VA-ECMO implantation, in which the increase in serum creatinine was more than 3.0 times baseline or increased to more than 4.0 mg/dL, or the patient required continuous renal replacement therapy (CRRT). The indications for initiation of CRRT included volume overload, progressive metabolic acidosis (pH <7.1~7.2 or serum bicarbonate level <12~15 mmol/L), and severe electrolyte abnormalities (potassium level >6.5 mmol/L, hyponatremia, hypernatremia, and hyperphosphatemia). Liver dysfunction was defined as the increase of total bilirubin in serum greater than 171 μmol/L, or an international normalized ratio greater than 1.5, or prothrombin coagulative activity lower than 40%. A cerebrovascular accident was diagnosed as cerebral hemorrhage or cerebral embolism based on cranial CT scans. Malignant arrhythmias were defined as ventricular arrhythmias that caused hemodynamic disturbances in a short period of time and required defibrillation or medication to restore sinus rhythm.

### 2.6 Statistics

The data are expressed as mean ± standard deviation (SD) in continuous variables with Gaussian distributions and n (%) in categorical variables. For the skewed variables, the median (interquartile range, IQR) was used. The student’s *t* test was used for investigating the impact of AKI on ICU stay, hospital stay, and mechanical ventilation time. Potential risk factors for developing AKI ≥3 were evaluated by the univariate logistic regression analysis. Candidate risk factors (*p *< 0.1) in the univariate analysis were entered into the multivariable logistic regression model. The tolerance and variance inflation factors (VIF) were calculated to evaluate the collinearity of the potential variables. When the tolerance <0.1 or the VIF >10, there is collinearity among the variables. A *p*-value of <0.05 was set for statistical significance. All of the analyses were performed using the SPSS statistics software (version 26.0, IBM, Corp., Armonk, NY, USA).

## 3. Results

### 3.1 Study Population

A total of 136 patients with a mean age of 53.6 ± 13.9 years (66.9% male) were identified for the study after excluding 62 patients undergoing a heart transplantation. Demographic characteristics of the study population is summarized in Table 1. The median time of ECMO support was 114.3 (44.1, 150.5) hours. VA ECMO was initiated in the operation room in 95 patients and in the intensive care unit (ICU) in 41 patients. Blood products were transfused in 81 patients intraoperatively and 125 patients postoperatively. 16 patients received central cannulation; the other 120 patients received peripheral cannulation. 80 patients (58.8%) developed AKI ≥3. CRRT was used in 93 patients, including all the AKI ≥3 patients and 13 patients of AKI <3. 36 patients recovered after the treatment of CRRT, 34 in the AKI ≥3 group.

**Table 1.** **Demographics of the study population**.

Variables		No. of patients	Variables	No. of patients
Age, year, Mean ± SD		53.6 ± 13.9	ECMO cannulation	
Gender, male, n (%)		91 (66.9)	Central cannulation, n (%)	16 (11.8)
BMI, kg/$m^{2}$, Mean ± SD		24.2 ± 4.2	Peripheral cannulation, n (%)	120 (88.2)
Charlson Comorbidity Index			Special Treatment	
	0, n (%)	48 (35.3)	CRRT, n (%)	93 (68.4)
	1, n (%)	28 (20.6)	ECMO time, hour, Median (IQR)	114.3 (44.1, 150.5)
	2, n (%)	29 (21.3)	Intraoperative transfusion, n (%)	81 (59.6)
	3, n (%)	23 (16.9)	Postoperative transfusion, n (%)	125 (91.9)
	4, n (%)	7 (5.1)	Ventilation time, hour, Median (IQR)	153.0 (27.5, 436.6)
	5, n (%)	1 (0.7)	Outcomes	
Type of Surgery			AKI 1, n (%)	16 (11.8)
	CABG, n (%)	30 (22.1)	AKI 2, n (%)	18 (13.2)
	Valvular Surgery, n (%)	33 (24.3)	AKI ≥3, n (%)	80 (58.8)
	Aortic arch surgery, n (%)	13 (9.6)	Septicemia, n (%)	25 (18.4)
	CABG + Valvular Surgery, n (%)	18 (13.2)	Pneumonia, n (%)	35 (25.7)
	Congenital heart operation, n (%)	18 (13.2)	Atrial fibrillation, n (%)	57 (41.9)
	CABG + Aortic arch surgery, n (%)	11 (8.1)	Liver dysfunction, n (%)	30 (22.1)
	Others*, n (%)	13 (9.6)	Malignant arrhythmia, n (%)	71 (52.2)
	ICU Stay, hour, Median (IQR)	264 (132, 540)	Cerebrovascular accident, n (%)	11 (8.1)
	Hospital Stay, day, Median (IQR)	22.4 (9.4, 41.4)	In-hospital Death, n (%)	81 (59.6)

* Pulmonary Endarterectomy, Pericardiectomy, Morrow, and Ventricular aneurysm surgery. AKI, acute kidney injury; BMI, body mass index; CABG, coronary artery bypass grafting; CRRT, continuous renal replacement therapy; ECMO, extracorporeal membrane oxygenation; ICU, intensive care unit; IQR, interquartile range.

### 3.2 Prognostic Impact of AKI ≥3

Complications that VA ECMO patients experienced included infections, and involved the circulatory, digestive and other systems. 18.4% (25/136) of patients suffered septicemia. 25.7% (35/136) patients developed pneumonia. 52.2% (71/136) patients experienced malignant arrhythmias and 57 patients had atrial fibrillation. Laboratory tests showed that 30 patients had liver dysfunction, and 8.1% (11/136) of patients suffered a cerebrovascular accident. The in-hospital mortality was 59.6% (81/136) (Table 1). Patients were grouped according to the occurrence of AKI ≥3. 80 (58.8%) patients developed AKI ≥3 according to the KDIGO stage 3. Binary logistic regression results showed that there was no significant difference in the in-hospital mortality between the two groups (62.5% vs. 57.5%, *p* = 0.559). Patients with AKI ≥3 required significantly longer mechanical ventilation (200.9 [128.0, 534.8] hours *vs.* 78.9 [13.0, 233.0] hours, *p *< 0.001). The ICU stay and hospital stay of patients with AKI ≥3 were significantly longer than that of patients with AKI <3 (384 [182, 648] hours *vs.* 216 [48, 456] hours, *p* = 0.001; 25.0 [15.3, 46.6] days *vs.* 13.4 [7.4, 38.4] days, *p* = 0.022, respectively).

### 3.3 Risk Factors for AKI ≥3

To investigate the potential risk factors for AKI ≥3, demographic characteristics, the Charlson Comorbidity index, preoperative laboratory tests, intraoperative factors and variables at the time of VA ECMO implantation were entered into the univariate analysis. There was no difference among all the demographic and preoperative factors between the two groups (Table 2). Before ECMO implantation, the mean arterial blood pressure of the AKI ≥3 group was lower than that of the AKI <3 group (58 ± 13 *vs.* 65 ± 14 mmHg, *p* = 0.020) (Table 3). The time of ECMO implantation (intraoperatively or postoperatively) was significantly different between the two groups (82.1% *vs.* 61.3%, *p* = 0.011). Previous studies have reported that age, lactate and ejection fraction (EF) may be risk factors for the poor outcome of patients receiving VA ECMO after PCS [6, 8]. Age, cross-clamp time, CPB time, the timing of ECMO implantation, mean artery pressure (MAP), lactate concentration before ECMO, and preoperative EF were entered into multivariable logistic regression analyses. The results showed that the timing of ECMO implantation was an independent risk factor for the development of AKI ≥3 (*p* = 0.036) (Table 4). Intraoperative implantation of ECMO may decrease the risk of AKI ≥3 (OR = 0.298, 95% CI = 0.096–0.925). The tolerance and variance inflation factors showed that there was no collinearity among these variables.

**Table 2.** **Preoperative factors for AKI ≥3**.

Variables		AKI <3	AKI ≥3	OR	95% CI	*p*
Age, year, Mean ± SD		55.1 ± 13.9	52.6 ± 14.0	0.987	(0.963, 1.012)	0.323
Gender, male, n (%)		40 (71.4)	50 (62.5)	1.640	(0.779, 3.454)	0.193
BMI, kg/$m^{2}$, Mean ± SD		24.2 ± 3.8	24.3 ± 4.5	1.008	(0.929, 1.093)	0.856
Charlson Comorbidity index						
	0, n (%)	22 (39.3)	26 (32.5)	Reference		
	1, n (%)	8 (14.3)	20 (25.0)	2.115	(0.780, 5.735)	0.141
	2, n (%)	14 (25.0)	15 (18.8)	0.907	(0.360, 2.283)	0.835
	3, n (%)	8 (14.3)	15 (18.8)	1.587	(0.567, 4.439)	0.379
	4, n (%)	3 (5.4)	4 (5.0)	1.128	(0.228, 5.594)	0.883
	5, n (%)	1 (1.8)	0 (0)	0.0	(0, 0)	>0.990
	EF, %, Median (IQR)	58 (53, 63)	60 (55, 64)	1.030	(0.991, 1.071)	0.137
Preoperative Laboratory test						
	CK, U/L, Median (IQR)	62 (48, 86)	58 (47, 84)	1.003	(0.995, 1.011)	0.443
	PTA, %, Median (IQR)	91 (78, 98)	92 (74, 101)	1.000	(0.984, 1.016)	0.987
	ALT, U/L, Median (IQR)	19 (14, 30)	23 (14, 32)	0.999	(0.980, 1.019)	0.951
	AST, U/L, Median (IQR)	23 (18, 30)	24 (18, 29)	1.002	(0.970, 1.034)	0.917
	CRP, mg/L, Median (IQR)	2.10 (0.66, 4.63)	1.67 (0.80, 3.63)	0.948	(0.838, 1.072)	0.394
	TB, μmol/L, Median (IQR)	15.90 (11.30, 25.22)	17.92 (11.65, 27.98)	1.006	(0.979, 1.034)	0.650
	DB, μmol/L, Median (IQR)	3.63 (2.38, 6.70)	4.28 (2.34, 6.76)	1.008	(0.926, 1.097)	0.856
	TG, mmol/L, Median (IQR)	1.33 (1.02, 2.16)	1.06 (0.88, 1.32)	0.768	(0.503, 1.172)	0.220
	Albumin, g/L, Median (IQR)	39.5 (38.5, 42.8)	40.6 (38.4, 42.9)	0.984	(0.906, 1.067)	0.692
	Platelet, ×${\mathrm{10}}^{9}$/L, Mean ± SD	188.8 ± 54.0	176.9 ± 70.2	0.997	(0.992, 1.002)	0.290
	HDL-C, mmol/L, Median (IQR)	1.03 (0.89, 1.24)	1.06 (0.88, 1.32)	1.706	(0.514, 5.665)	0.383
	LDL-C, mmol/L, Median (IQR)	2.47 (1.89, 3.05)	2.23 (1.74, 2.82)	0.716	(0.463, 1.106)	0.132
	Creatinine, μmol/L, Median (IQR)	82.3 (72.0, 94.8)	86.4 (69.8, 98.1)	0.996	(0.983, 1.010)	0.605

ALT, alanine aminotransferase; AST, aspartic transaminase; BMI, body mass index; CK, creatine, kinase; CI, confidence interval; CRP, C-reaction protein; DB, direct bilirubin; EF, ejection fraction; HDL-C, high density lipoprotein cholesterol; LDL-C, low density lipoprotein cholesterol; MAP, mean artery pressure; PTA, Prothrombin activity; OR, odds ratio; RBC, red blood cell; TB, total bilirubin; TG, triglyceride; TP, total protein.

**Table 3.** **Intraoperative and ECMO-implantation associated factors for AKI ≥3**.

Variables		AKI <3	AKI ≥3	OR	95% CI	*p*
Intraoperative Factors						
	Transfusion rate, n (%)	30 (53.6)	51 (63.7)	1.524	(0.760, 3.055)	0.235
	CPB time, min, Median (IQR)	260 (137, 455)	236 (119, 351)	0.998	(0.996, 1.000)	0.025
	RBC transfusion, U, Median (IQR)	0 (0, 4)	0 (0, 4)	1.036	(0.949, 1.130)	0.433
	FFP transfusion, mL, Median (IQR)	200 (0, 700)	400 (0, 800)	1.000	(0.999, 1.001)	0.756
	Platelet transfusion, U, Median (IQR)	0 (0, 1)	0 (0, 1)	1.440	(0.892, 2.323)	0.135
	Cross-clamp time, min, Median (IQR)	100 (68, 184)	100 (55, 150)	0.996	(0.991, 1.000)	0.072
	Epinephrine, mg/kg/min, Mean ± SD	0.058 ± 0.08	0.061 ± 0.06	1.851	(0.013, 265.89)	0.808
	Dopamine, mg/kg/min, Mean ± SD	4.56 ± 2.83	4.77 ± 3.70	1.019	(0.918, 1.132)	0.719
	Norepinephrine, mg/kg/min, Mean ± SD	0.075 ± 0.27	0.064 ± 0.09	0.744	(0.121, 4.563)	0.179
Variables at VA ECMO Implantation						
	MAP, mmHg, Mean ± SD	65 ± 14	58 ± 13	0.965	(0.936, 0.994)	0.020
	Heart Beat, bpm, Mean ± SD	104 ± 27	104 ± 23	1.001	(0.980, 1.022)	0.946
	Lactate, mmol/L, Mean ± SD	8.3 ± 6.6	10.3 ± 4.7	1.068	(0.989, 1.154)	0.095
	Intraoperative ECMO, n (%)	46 (82.1)	49 (61.3)	0.344	(0.152, 0.779)	0.011
	Peripheral cannulation, n (%)	52 (92.9)	68 (85.0)	0.436	(0.133, 1.430)	0.171
	Speed, rpm, Mean ± SD	3224 ± 422	3145 ± 389	1.000	(0.998, 1.001)	0.403
	Flow rate, L/min, Mean ± SD	2.9 ± 0.6	3.0 ± 0.6	1.000	(1.000, 1.001)	0.392

AKI, acute kidney injury; CABG, coronary artery bypass grafting; CI, confidence interval; CPB, cardiopulmonary bypass; FFP, fresh frozen plasma; MAP, mean arterial pressure; ICU, intensive care unit; OR, odds ratio; RBC, red blood cell; VA ECMO, venoarterial extracorporeal membrane oxygenation.

**Table 4.** **Multivariable logistic regression analysis of AKI ≥3**.

Variables	β	OR	95% CI	*p*	Tolerance	VIF
Age	0.000	1.000	(0.962, 1.038)	0.984	0.870	1.150
CPB time	0.000	1.000	(0.997, 1.003)	0.976	0.701	1.427
Cross-clamp time	–0.004	0.996	(0.989, 1.003)	0.242	0.706	1.416
EF preoperatively	0.044	1.045	(0.987, 1.106)	0.128	0.893	1.119
Early ECMO implantation*	–1.210	0.298	(0.096, 0.925)	0.036	0.829	1.206
MAP before ECMO implantation	–0.024	0.976	(0.942, 1.012)	0.194	0.879	1.138
Lactate before ECMO implantation	–0.002	0.966	(0.907, 1.098)	0.998	0.845	1.184

*Implantation of ECOM in operation room *vs.* Implantation of ECMO in intensive care unit. CI, confidence interval; CPB, cardiopulmonary bypass; ECMO, extracorporeal membrane oxygenation; EF, ejection fraction; MAP, mean arterial pressure; OR, odds ratio; VIF, variance inflation factor.

## 4. Discussion

To evaluate the incidence and impact of AKI ≥3 in patients with PCS supported by VA ECMO, we conducted this retrospective observational study and found that 58.8% of patients developed AKI ≥3 (KDIGO stage 3). In this study population, patients with AKI ≥3 required longer mechanical ventilation support. The length of ICU stay and hospital stay were significantly prolonged. Furthermore, we found that the risk factor for developing AKI ≥3 was the timing of VA ECMO implantation. Compared with postoperative implantation, intraoperative implantation of VA ECMO can be a protective factor to prevent AKI ≥3.

A previous meta-analysis found that the incidence of AKI during ECMO treatment was 72% and the incidence of AKI ≥3 was 50% [9]. The incidence of AKI ≥3 in postcardiac surgery patients supported by VA ECMO is higher, 65.0~65.7% [5, 7]. Our data is consistent with previous findings with a rate of 58.8%, which suggests that patients with PCS have a higher incidence of AKI ≥3. Several factors contribute to this observation. The low cardiac output during PCS decreases the level of organ perfusion and results in kidney dysfunction. In addition, frequent use of intravenous fluid and inotropes and vasopressors can result in a renal ischemia-reperfusion injury. It is a key pathogenic event in the microcirculatory dysfunction leading to AKI [10]. It has been reported that older age was associated with poor prognosis in coronary artery bypass grafting patients who received VA ECMO [11]. Patients in our cohort were younger than in other studies, which may explain why our incidence was a slightly lower than in previous studies [5, 7].

In 2021, a retrospective analysis demonstrated that mortality was increased in those patients who developed AKI during VA ECMO after cardiac surgery [12]. However, in our study the in-hospital mortality of AKI ≥3 patients was similar to that of AKI <3 patients. In our center, nearly all AKI ≥3 patients received aggressive treatment, such as CRRT, which could have improved patient outcomes. Many complications occurred during the management of VA ECMO, which contributes to prolonged ventilation time, ICU stay, and hospital stay. Compared with AKI <3 patients, the duration of hospital treatment was significantly longer in AKI ≥3 patients.

The etiology of AKI after ECMO treatment is related to prerenal and renal factors, such as red cell distribution width, baseline left ventricular ejection fraction, and serum lactate levels at the initiation of ECMO [6, 13]. Before surgery, all cardiac surgery patients are evaluated for abnormalities in renal function. Our data showed that the demographic characteristics and intraoperative factors were not associated with the occurrence of AKI ≥3, but the timing of ECMO implantation were significantly different between the two groups. Intraoperative implantation of ECMO was associated with a decreased incidence of AKI ≥3, which was inconsistent with a previous study [7]. The risk factors for AKI in patients with acute respiratory failure supported by veno-venous ECMO did not include the timing of ECMO implantation [14]. These results indicate that there were some potential factors in patients with PCS that increased the risk of renal injury before ECMO implantation. The most likely factor was cardiac output. In patients undergoing PCS for a long period, low cardiac output reduces renal perfusion and oxygen delivery, and subsequently resulted in kidney injury [15]. Intraoperative implantation limits the duration of low cardiac output and helps to avoid organ dysfunction [7]. Therefore in patients with PCS, the timing of ECMO implantation should be chosen to avoid prolonging the duration of low cardiac output. This may help decrease the risk of AKI ≥3 during ECMO.

Our study has several limitations. Undetected or identified confounders may have biased the regression analysis because of its retrospective and single center design. The complications were described in Table 1. However, we could not assess the timing of each complication. Hence, we could not investigate whether these complications are risk factors or outcomes for AKI ≥3. In the subsequent data extraction, it is necessary to pay attention to the time of occurrence of the various complications. This study lacks follow-up data for the surviving patients. Nonetheless, the risk factors we identified were consistent with those described in previous studies.

## 5. Conclusions

In summary, the incidence of AKI ≥3 of patients supported by VA ECMO after PCS was 58.8% in our center. Patients with AKI ≥3 required significantly longer mechanical ventilation and hospital stay. Intraoperative implantation of ECMO was associated with a decreased incidence of AKI ≥3. Intraoperative implantation of VA ECMO can be a protective factor for preventing AKI ≥3.

## Data Availability

The data presented in this study are available on request from the corresponding author.

## Supplementary Material

Supplementary material associated with this article can be found, in the online version, at https://doi.org/10.31083/j.rcm2403091.
