# Supplementary material for: Evaluation of Acute Kidney Injury in Postcardiotomy Cardiogenic Shock Patients Supported by Extracorporeal Membrane Oxygenation
Source: Rev Cardiovasc Med. 2023 Mar 16;24(3):91. doi: 10.31083/j.rcm2403091 (PMC11264042; doi:10.31083/j.rcm2403091)
Supplement: Supplementary file 1 [file 2153-8174-24-3-091-s1.docx]

Evaluation of Acute Kidney Injury in Postcardiotomy Cardiogenic Shock Patients Supported by Extracorporeal Membrane Oxygenation

**Anticoagulation Management of VA ECMO.**

The ECMO system was implanted under the condition in which the activated clotting time (ACT) was kept longer than 410 seconds with the unfractionated heparin. When the hemodynamic parameters were stable, the patients were given continuous heparization to maintain an ACT of 180 ~ 220 seconds or activated partial thromboplastin time of 50 ~ 70 seconds. The goal after full ECMO support was to reduce intravenous inotropes to allow optimal myocardial recoveries. Cardiopulmonary recovery was assessed daily by clinical, echocardiographic, and hemodynamic measurements to define the optimal weaning time.
